# Expression of circulating GRP78 and gp96, endoplasmic reticulum stress-related proteins, in menstrual and bladder pain sensitivity

**DOI:** 10.1038/s41598-026-58824-0

**Published:** 2026-06-30

**Authors:** Chandrashekara N. Kyathanahalli, Sarah E. Darnell, Frank F. Tu, Kevin M. Hellman

**Affiliations:** 1 Department of Obstetrics and Gynecology, Endeavor Health, Evanston, IL USA; 2 Department of Obstetrics and Gynecology, Pritzker School of Medicine, University of Chicago, Chicago, IL USA

**Keywords:** Dysmenorrhea, Bladder pain, Heat shock proteins, Systemic circulation, Biomarkers, Diseases, Medical research, Risk factors

## Abstract

Dysmenorrhea is often linked to uterine inflammation, but the additional factors and pathways that contribute to its pathophysiology remain poorly understood. Given growing evidence linking endoplasmic reticulum (ER) stress to inflammation and pain sensitization, we examined circulating ER stress-associated heat shock proteins (HSPs) GRP78 and gp96 in individuals with dysmenorrhea (n = 82), a dysmenorrhea subtype with bladder pain sensitivity (DYSB, n = 26) previously shown to increase risk for chronic pain, and controls (n = 19). After correcting for Menstrual phase, naproxen exposure, and oral contraceptive use, GRP78 was higher in DYSB than in DYS (ratio 1.33; *p* = .028), while gp96 was lower in both DYS (ratio 0.62; *p* = .019) and DYSB (ratio 0.59; *p* = .031) compared to controls. gp96 was also lower during the menstrual phase with naproxen compared to the non-menstrual phase (ratio 0.73; *p* = .014), whereas GRP78 was not significantly affected by menstrual phase, naproxen, or oral contraceptive use, and neither protein was associated with anxiety, depression, or sleep disturbance scores. These cross-sectional findings suggest that dysmenorrhea, particularly the bladder-sensitive subtype (DYSB), is associated with divergent circulating levels of two ER stress-related proteins. The inverse pattern of higher GRP78 and lower gp96 levels points to selective, rather than global, ER chaperone dysregulation as a candidate mechanism. However, future mechanistic and longitudinal investigations will be required to establish whether GRP78 and gp96 carry predictive or pathophysiological relevance.

**Supplementary Information:**

The online version contains supplementary material available at https://doi.org/10.1038/s41598-026-58824-0.

## Introduction

Dysmenorrhea, moderate-to-severe intensity cramping pain preceding or accompanying menstruation, affects 50% or more of reproductive-age individuals assigned female at birth worldwide^1–5^ and is a leading risk factor for the development of chronic pain^6–9^. Although dysmenorrhea is historically attributed to local uterine inflammation^14–16^, the incomplete efficacy of nonsteroidal anti-inflammatory drugs (NSAIDs)^17^ and emerging evidence of peripheral immune alterations and elevated inflammatory cytokines^15,18–20^ suggest that systemic immune mechanisms beyond local uterine inflammation may contribute to the pathophysiology of dysmenorrhea and heightened chronic pain risk.

One potential pathway connecting systemic inflammation in dysmenorrhea to chronic pain risk is cellular stress signaling, particularly endoplasmic reticulum (ER) stress, which can amplify immune activation. In preclinical models, ER stress can sensitize peripheral nociceptors (e.g., by lowering activation thresholds in dorsal root ganglion neurons)^21^. Under physiological conditions, the endoplasmic reticulum maintains protein homeostasis by regulating protein folding, maturation and trafficking, while preventing the accumulation of misfolded or unfolded proteins^22^. However, conditions such as hypoxia, ischemia, or nutrient deprivation can overwhelm the folding capacity, leading to the accumulation of misfolded proteins within the lumen, resulting in ER stress. In response, the cell activates the unfolded protein response (UPR), which reduces global protein synthesis and upregulates heat shock proteins (HSPs) to restore proteostasis. If ER stress persists and resolution fails, the UPR triggers the release of inflammatory cytokines, which are major contributing factors to the pathogenesis of neurodegenerative and metabolic diseases^23,24^. Animal models of spinal nerve ligation or cancer induction with pain show ER stress and subsequent UPR activation contribute to peripheral nociceptor sensitization^21,25,26^. In these models, ER stress promotes peripheral nociceptor hyperexcitability and lowers nociceptive thresholds through modulating ion channel activity and enhancing release of pro-inflammatory mediators^27–30^. Although it is unknown whether abnormal ER stress and UPR activation occurs in patients with primary dysmenorrhea, in endometriosis (a classic secondary dysmenorrhea disorder), there is increased expression of markers of ER stress in endometrial cells such as p-IRE, and HSP GRP78^31–35^.

To examine whether ER homeostasis is disrupted in individuals with dysmenorrhea, we focused on two stress-inducible HSPs: Glucose-regulated proteins of 78 kDa (GRP78, also HSPA5 or BiP) and 94 Da (GRP94, also HSP90B1 or gp96)^36^. These proteins were selected based on growing evidence linking them to pain pathways across several conditions, including sciatica^37^, diabetic neuropathy^38^, rheumatoid arthritis^39^ and pain related to stroke ^40^ or bone cancer^41^. In a mouse model of dysmenorrhea, endometrial expression of HSP90ab1 (an isoform of gp96) and cyclooxygenase-2 (COX-2) was increased, and inhibition of HSP90ab1 with geldanamycin reduced COX-2 expression and alleviated pain behavior ^42^. Beyond their canonical intracellular chaperone roles, both GRP78 and gp96 can be secreted extracellularly, where they influence immune responses and inflammation ^42–45^, mechanisms that underlie pain-related conditions ^46–51^. Notably, serum HSP90ab1 is elevated in young adolescents diagnosed with endometriosis^52^. Despite these observations, the roles of gp96 and more so GRP78 in pain remain understudied, especially menstrual pain^53^.

The present study evaluates individuals with clinically bothersome menstrual pain (dysmenorrhea) and controls. Among those with dysmenorrhea, there is a subtype that exhibits bladder pain sensitivity during experimental testing (DYSB) even before the development of chronic pain^10,11^. Although dysmenorrhea is associated with chronic pain, this experimentally defined subtype, DYSB, may confer greater chronic pain risk than dysmenorrhea alone^12,13^. Given the elevated risks in this cohort, it is important to establish the mechanisms underlying the progression to daily non-menstrual pelvic pain for the prevention of chronic pelvic pain. Throughout this manuscript, we distinguish three levels of pain-related phenomena: nociceptive processing (peripheral detection and transmission of noxious stimuli, typically inferred from preclinical models and electrophysiological measures); experimentally assessed pain sensitivity (altered response thresholds measured with quantitative sensory tests, such as the bladder distension task used here to identify DYSB); and clinical pain experience (self-reported pain intensity, such as menstrual cramps).

Given this prior research, we hypothesized that circulating levels of GRP78 and gp96 would be increased in individuals experiencing menstrual pain, with a more pronounced increase in those also exhibiting bladder pain sensitivity (DYSB), who appear at higher risk for chronic pelvic pain later in life than even dysmenorrhea alone^12^. Building on the evidence that inflammatory changes persist across the menstrual cycle in dysmenorrhea^18,19^, we further hypothesized that serum GRP78 and gp96 levels would remain stable across menstrual phases. While the primary analgesic effects of NSAIDs are attributed to the suppression of prostaglandin synthesis, some evidence suggests that HSPs may also contribute to their analgesic mechanisms^54–56^ and that HSP concentrations are correlated to pain symptom intensity^57–59^. Therefore, we also conducted an exploratory analysis of whether treatment with naproxen sodium reduces circulating GRP78 and gp96 content. Finally, we examined associations between GRP78/gp96 and clinical covariates and symptoms to characterize how these circulating proteins relate to clinical features of dysmenorrhea and DYSB, an experimentally defined subphenotype that may confer greater chronic pelvic pain risk than dysmenorrhea alone.

## Materials and methods

### Study type

We conducted a secondary analysis using data from three independent research studies investigating uterine physiology and the effectiveness of NSAIDs in the management of dysmenorrhea (Supplementary Fig. S1)^16,60^. All protocols were approved by the Institutional Review Board of Endeavor Health (formerly NorthShore University HealthSystem) [IRB approval EH19-040, EH18-128, EH16-263], and conducted in accordance with relevant guidelines and regulations (including the Declaration of Helsinki). The manuscript does not include images or other information that could identify individual participants. Written informed consent was obtained from all participants before enrollment. Participants completed a screening questionnaire and maintained menstrual diaries for at least one full menstrual cycle that documented physical and psychological symptoms, as well as current medication use. Participants also filled out a 19-item body map indicating where they had pain over the past week. We administered a battery of validated PROMIS questionnaires, each using a Likert scale (never, rarely, sometimes, often, always), to assess symptoms of anxiety (Short Form 7a), depression (Short Form 8b), and sleep disturbance (Short Form 8b)^61^.

Participants were categorized into three groups: those with dysmenorrhea, those with dysmenorrhea and comorbid bladder pain sensitivity (DYSB), and controls. Individuals in the dysmenorrhea group reported moderate-to-severe menstrual pain, defined as > 4 on a 0–10 numerical rating scale (NRS). Participants in the DYSB group met the criteria for dysmenorrhea and also reported bladder pain rating > 15 on a 0–100 visual analog scale (VAS) upon reaching first urge or within 1 h, whichever came first, during a rapid version of our bladder distension task^10,62^. Briefly, participants consumed a standardized volume of water (0.59 L) and rated bladder pain and urgency on a 0–100 VAS at first urge to void or after a maximum of one hour, whichever occurred first. The same protocol and classification threshold (VAS > 15) were applied uniformly across all three cohorts. Controls reported average menstrual pain < 3/10. These pain thresholds were specifically chosen based on prior cluster analyses and thresholds for increased risk for non-menstrual pain^9,10^.

#### Study #1: noninvasive imaging of uterine physiology to improve treatment for dysmenorrhea

This study employed magnetic resonance imaging (MRI) to investigate the uterine mechanisms in menstrual pain in individuals of reproductive age (18–45 years, See Supplementary Table S2 for detailed demographics for each study)^60^. Each participant completed two study visits—one during menstruation and one during the non-menstrual phase (16–25 days after menses). To minimize pharmacological interference, participants were instructed to abstain from all pain medications for at least 8 h prior to visit. Blood samples were collected during both sessions.

#### Study #2: mechanistic characterization of uterine pain (MCUP) to improve diagnosis and treatment for dysmenorrhea

The goal of MCUP was to investigate the physiological mechanisms underlying menstrual pain and the effects of NSAIDs on these mechanisms within a randomized, placebo-controlled study (NCT04145518). Participants aged 18–45 were required to have a menstrual flow and abstain from medication before the visit. After an MRI, participants received naproxen sodium or a placebo. Blood samples were obtained 90 min post-treatment.

#### Study #3: examining the role of improved NSAID management in treating dysmenorrhea

The objective of this study (NCT03697720) was to determine whether optimized use of naproxen sodium relieves menstrual pain and subsequent chronic pelvic pain risk. Baseline visits were scheduled during the luteal phase (15 to 25 days after the onset of the previous menstrual cycle). At the time of the visit, participants confirmed that menstrual bleeding or pain had not yet begun.

Participants were instructed to take a naproxen sodium tablet (550 mg) at home on the morning before they anticipated menstruation. They were asked to take the second dose 12 h later. This regimen was continued for the next three days of menstruation. Participants were asked to follow this dosing schedule before and during menses for 6–8 months until their follow-up visit. This visit was scheduled during menstruation and blood samples were collected 90 min after ingestion of a naproxen sodium tablet.

### Inclusion and exclusion criteria

Participants across all study cohorts were required to be English-speaking and have regular menstrual cycles. Participants were excluded if they met any of the following conditions: a) the presence of active pelvic or abdominal malignancies (primary or metastatic), b) absence of regular menses (including current pregnancy, recent pregnancy, or active breastfeeding), c) active genitourinary infection, d) unwillingness or inability to comply with study procedures, e) diagnosis of hypertension or increased risk for hypertension, f) contraindications to naproxen sodium use, including allergies, kidney disease, anemia, alcoholism, cardiovascular disease, gastro-intestinal disorders, g) use of hormonal medications or diagnosis of polycystic ovarian syndrome, h) MRI study-specific exclusion: the presence of intrauterine devices (alters local MRI signal), history of metallic implants/injury, and conditions that preclude investigation with MRI, i) relevant comorbidities such as thyroid or adrenal dysfunction, renal or hepatic disorders, genitourinary infection, and, known pelvic pathology, including prior clinical or imaging-based diagnosis of endometriosis, adenomyosis, uterine fibroids, ovarian cysts, or pelvic inflammatory disease, j) body mass index (BMI) greater than 40. Screening for these exclusionary conditions was based on self-reported medical history, review of available medical records, and transvaginal ultrasound where previously performed. Definitive imaging (pelvic MRI) or laparoscopic confirmation of the absence of endometriosis was not obtained in every participant; subclinical endometriosis therefore cannot be fully excluded and is acknowledged as a limitation.

### Blood samples and serum separation

Blood samples were obtained via antecubital venipuncture and collected into serum-separating vacutainer tubes (BD and Co., Franklin Lakes, NJ). See Supplementary Fig. S1 for information on how each sample was collected during menses or nonsenses. A summary of visit timing, menstrual phase, and NSAID exposure across the three cohorts is provided in Supplementary Table S1. Within 120 min of collection, samples were centrifuged at 3000 rpm (Eppendorf, 5810R) for 10 min at room temperature. The resulting serum was separated and stored at − 80 °C until analysis.

### Quantification of GRP78 and gp96 by ELISA

Serum concentrations of GRP78 (HSPA5) and gp96 (HSP90B1) were measured using commercially available enzyme-linked immunosorbent assay (ELISA) kits (GRP78: ADI-900–214, Enzo Life Sciences; gp96: NBP2-76,451, Novus Biologicals), following manufacturer protocols. The sensitivity range of the GRP78 assay was 8.4–4500 ng/ml and 0.19–20 ng/ml for gp96.

Serum was diluted 1:30 times in assay buffer for GRP78 analysis, but not diluted for gp96. A total of 100 µL of each sample was loaded per well. Concentrations were determined based on four-parameter logistic (4PL) standard curves (GRP78: R^2^ = 0.998; gp96: R^2^ = 0.999). The intra-assay and inter-assay coefficients of variation (CV) were as follows: GRP78 (5%, 10%) and gp96 (5%, 15%). Six samples were excluded due to hemolysis or centrifugation protocol deviations; no additional samples were excluded beyond these six. The ~ 15% inter-assay CV for gp96 falls within the ≤ 20% threshold widely accepted for ELISA-based quantification of low-abundance circulating proteins but is noted as a limitation^63^.

### Data and statistical analyses

All statistical analyses were conducted using R (version 4.4.3). Because multiple samples were obtained in different conditions (menses, non-menses, naproxen, naproxen-free), we fit linear mixed-effects models to simultaneously account for repeated measures within participants, menstrual phase, naproxen exposure, and oral contraceptive use. GRP78 and gp96 distributions were right-skewed and residual variance increased with the mean (visual inspection of residual plots; Shapiro–Wilk *p* < 0.05 on raw values). Log-transformation produced approximately normal distributions with homogeneous residual variance and yielded model coefficients interpretable as ratios of geometric means. The primary model was log(outcome) ~ Dysmenorrhea_Status + DYSB_Status + condition (non-menses, menses-naproxen-free, menses-naproxen) + Oral_Contraceptive_Use + BMI + (1 | participant). The participant-level random intercept accounts for within-subject correlation across repeated observations. BMI was included a priori because circulating heat shock proteins are known to vary with body mass index^64,65^; a preliminary analysis confirmed a significant BMI effect on GRP78 in this cohort. The DYSB vs Control contrast was derived as the sum of the Dysmenorrhea_Status and DYSB_Status coefficients. Models were fit in R using the lme4 package with REML estimation; p-values for fixed effects were computed using Satterthwaite’s approximation via lmerTest. Prior to primary analysis, observations with standardized residuals exceeding |z|> 3 were identified as outliers and excluded: two GRP78 observations met this criterion (subjects 44 and 50; 8,675 and 8,005 ng/mL, respectively). No gp96 observations exceeded this threshold. Pairwise group contrasts (DYS vs Control, DYSB vs DYS, DYSB vs Control) were computed for each protein separately, with Benjamini–Hochberg correction applied across the three comparisons within each protein^66^. Menstrual phase and naproxen effects were evaluated as separate fixed effects within the same mixed model, preserving within-subject structure. Given the small (n = 21) heterogeneous pooled dataset and sparse cell structure, conclusions about phase stability and naproxen effects should be considered explicitly exploratory. Two pre-specified sensitivity analyses were performed: (a) a within-study z-score sensitivity analysis, in which log(outcome) was standardized within each cohort before modeling, to verify that the primary effects were not driven by between-cohort scale differences (Supplementary Table S3); and (b) a paired within-subject phase analysis restricted to subjects with both menstrual and non-menstrual observations (n = 21). Full analysis code and the de-identified analysis dataset are available in our Open Science Framework repository (https://doi.org/10.17605/OSF.IO/EP3Y2). Statistical significance was defined as *p* < 0.05. Associations between GRP78 or gp96 and clinical or psychological covariates were evaluated on per-subject averages of log-transformed concentrations using Spearman’s rho for continuous covariates (age, number of painful body sites, and PROMIS Anxiety, Depression, Sleep Disturbance, and Fatigue scores), with Benjamini–Hochberg correction applied across 2 proteins × 6 covariates. We had 80% power to detect a Spearman correlation of r = 0.25.

Data are reported as medians with interquartile ranges (25th–75th percentiles), unless otherwise specified.

## Results

### Participant demographic, menstrual, systemic and psychological characteristics

The median ages of the participants in each group ranged from 23 to 28 years and were comparable across the groups (Table 1). The majority identified as White (62–79%) and non-Hispanic.

**Table 1:** Demographic and clinical characteristics of study participants across the three groups, dysmenorrhea, dysmenorrhea with comorbid bladder pain hypersensitivity (DYSB), and controls.

	Dysmenorrhea (n = 82)	DYSB (n = 26)	Control (n = 19)
Age (years)	24.5 [20, 30]	23 [21, 27]	28 [22, 33]
Race Asian White Black Multiple race Other Missing	8 (9.8%) 52 (63.4%) 13 (15.9%) 6 (7.3%) 2 (2.4%) 1 (1.2%)	4 (15.4%) 16 (61.5%) 5 (19.2%) 1 (3.8%) 0 (0%) 0 (0%)	3 (15.8%) 15 (78.9%) 1 (5.3%) 0 (0%) 0 (0%) 0 (0%)
Ethnicity Hispanic	21 (25.9%)	2 (7.7%)	2 (10.5%)
Average menstrual pain No NSAID (0–100 VAS)	76 [67, 82]	70 [66.2,84.5]	4 [0,13.5]
NSAID Use (0–100 VAS)	40.5 [25.5,60]	40.5[31.5,56.2]	4 [0,10]
Days of missed work/ school (last 90 days)	2 [0,3]	2 [0,3]	0 [0,0]
Menstrual cycle regularity Always regular Sometimes irregular Always irregular	69 (84.1%) 12 (14.6%) 1 (1.2%)	21 (80.8%) 2 (7.7%) 3 (11.5%)	18 (94.7%) 1 (5.3%) 0 (0%)
Oral contraceptive (current) No	67 (81.7%)	21 (80.8%)	11 (57.9%)
Diagnosed medical conditions CPP Diabetes IBD Migraine/Headache PBS/IC	2 (2.4%) 1 (1.2%) 0 (0%) 5 (6.1%) 0 (0%)	2 (7.7%) 1 (3.8%) 0 (0%) 3 (11.5%) 1 (3.8%)	0 (0%) 0 (0%) 1 (5.3%) 1 (5.3%) 0 (0%)
Body Mass Index (BMI, kg/m^2^)	24.4 [21.5, 27.4]	22.3 [20.8, 24.5]	23.5 [22.0, 26.9]

Data represented as median [IQR]. NSAID, Non-steroidal anti-inflammatory drugs; VAS, Visual Analog Scale; CPP, Chronic Pelvic Pain, IBD, Inflammatory Bowel Disease; BPS/IC, Bladder Pain Syndrome/Interstitial Cystitis.

As expected, participants in both the dysmenorrhea and DYSB groups reported occasional missed school or work and elevated average VAS menstrual pain scores over the past 90 days without analgesic use (76^67–82^ and 70^66–85^), with notable reductions following NSAID use. Current oral contraceptive use was most prevalent among controls (42.1%) and less common among those with dysmenorrhea (18.3%) or DYSB (19.2%). The prevalence of diagnosed medical conditions and median BMI values were similar across groups. Participant characteristics were consistent across the three contributing studies (Supplementary Table S2).

### Circulating GRP78 is higher in DYSB, while gp96 is lower in dysmenorrhea and DYSB

**Fig. 1:**
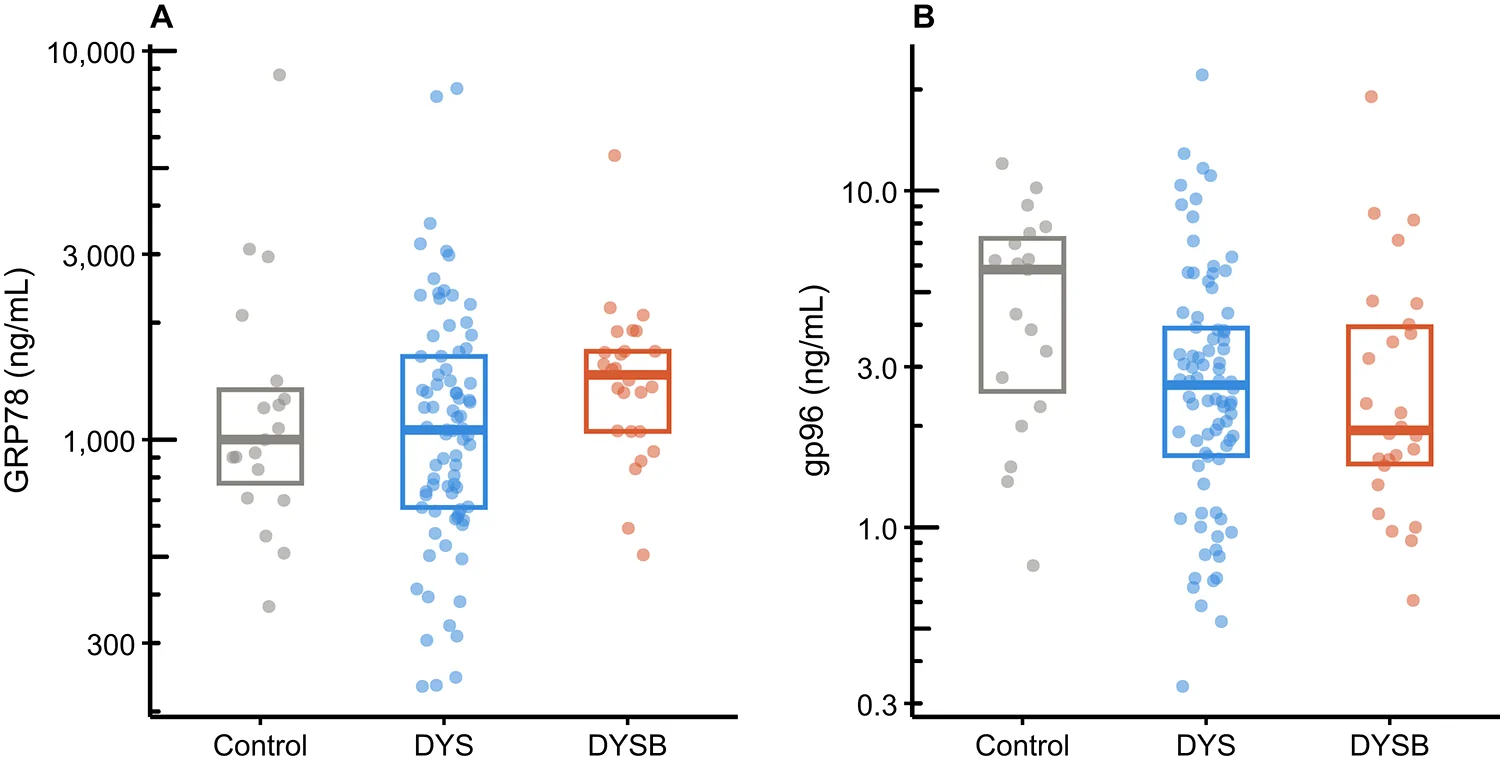
Circulating GRP78 and gp96 by diagnostic group. Subject-level geometric means (circles) with estimated marginal means (diamonds) and 95% confidence intervals from the primary linear mixed-effects model. (**A**) GRP78 in controls (n = 18), dysmenorrhea without bladder pain sensitivity (DYS, n = 80), and dysmenorrhea with bladder pain sensitivity (DYSB, n = 26); two GRP78 outliers (subjects 44 and 50, |z|> 3) excluded. The Bladder effect (DYSB vs. DYS) was significant (ratio 1.33 [1.03, 1.72]; *p* = .028). (**B**) gp96 in controls (n = 19), DYS (n = 82), and DYSB (n = 26). Both DYS (ratio 0.62 [0.41, 0.92]; *p* = .019) and DYSB (ratio 0.59 [0.37, 0.95]; *p* = .031) were significantly lower than controls. Y-axis: log10 scale. *p < 0.05.

Serum GRP78 and gp96 were detectable in all participants across all groups (Fig. 1). Circulating GRP78 was ~ 37% higher in DYSB participants than in those with dysmenorrhea alone (*p* = 0.014), whereas GRP78 levels in dysmenorrhea did not differ from controls (*p* = 0.821). Adjusting for menstrual phase, naproxen exposure, and oral contraceptive use, estimated GRP78 concentrations were 978 ng/mL in controls, 1011 ng/mL in dysmenorrhea, and 1389 ng/mL in DYSB (Table 2).

**Table 2:** Model effects. GRP78 analysis excludes two outliers (subjects 44 and 50, identified from full-sample model residuals |z|> 3). gp96 includes all subjects. DYS/B vs Control = dysmenorrhea effect (all DYS including DYSB). Bladder = additional effect of bladder sensitivity within DYS. Derived DYSB vs Control = β_DYS + β_Bladder. exp(β) = ratio of geometric means. M = Menses, NM = Nonmenses. OC use = Oral Contraceptive use * p < .05; ** p < .01.

	GRP78	GRP78	GRP78	GRP78	GRP78	gp96	gp96	gp96	gp96	gp96
Parameter	β	SE	t	p		β	SE	t	p	
DYS/B vs Control	0.034	0.148	0.23	0.821		− 0.468	0.203	− 2.30	0.023	*
Bladder: DYSB vs DYS	0.318	0.129	2.47	0.014	*	− 0.063	0.179	− 0.35	0.725	
Derived: DYSB vs Control	0.351	0.174	2.02	0.044	*	− 0.531	0.240	− 2.21	0.028	*
M (naproxen− free) vs NM	− 0.070	0.125	− 0.56	0.576		− 0.193	0.139	− 1.39	0.166	
M (naproxen) vs NM	− 0.129	0.121	− 1.07	0.286		− 0.322	0.130	− 2.48	0.014	*
OC use: Yes vs No	0.043	0.127	0.34	0.734		0.248	0.174	1.43	0.156	
BMI (per kg/m^2^)	0.034	0.013	2.64	0.009	**	− 0.021	0.018	− 1.22	0.225	

Conversely, circulating gp96 was ~ 40% lower in both dysmenorrhea (*p* = 0.023) and DYSB (*p* = 0.028) compared to controls, and gp96 levels in DYSB did not differ further from those in dysmenorrhea (*p* = 0.820). Adjusting for menstrual phase, naproxen exposure, and oral contraceptive use, estimated gp96 concentrations were 3.95 ng/mL in controls, 2.47 ng/mL in dysmenorrhea, and 2.32 ng/mL in DYSB (Table 2). Effect-size ratios and 95% confidence intervals for all contrasts are in Table 3; the within-study z-score sensitivity analysis supported the robustness of the gp96 findings across cohorts (Supplementary Table S3). The GRP78 DYSB effect was directionally consistent in the z-score sensitivity analysis but became borderline (p = 0.054), and should therefore be treated as preliminary pending replication.

**Table 3:** Estimated marginal means. Geometric means (ng/mL), marginal over phase, OC, and BMI (BMI evaluated at the sample mean). GRP78 excludes subjects 44 and 50 (residuals |z|> 3 in initial model). gp96 includes all subjects.

Group	GRP78 [95% CI]	gp96 [95% CI]
Control	978 [818, 1,170]	3.951 [3.106, 5.025]
DYS	1,011 [911, 1,123]	2.474 [2.168, 2.824]
DYSB	1,389 [1,196, 1,614]	2.323 [1.899, 2.842]

Given that GRP78 was higher and gp96 was lower in participants with pain, we asked whether circulating levels of the two proteins were coupled at the individual level. Across all participants, GRP78 and gp96 were inversely correlated (Spearman’s ρ =  − 0.327, p < 0.001, n = 124; Supplementary Fig. S2): individuals with higher GRP78 had lower gp96.

### GRP78 and gp96 levels are not influenced by menstrual phase

**Fig. 2:**
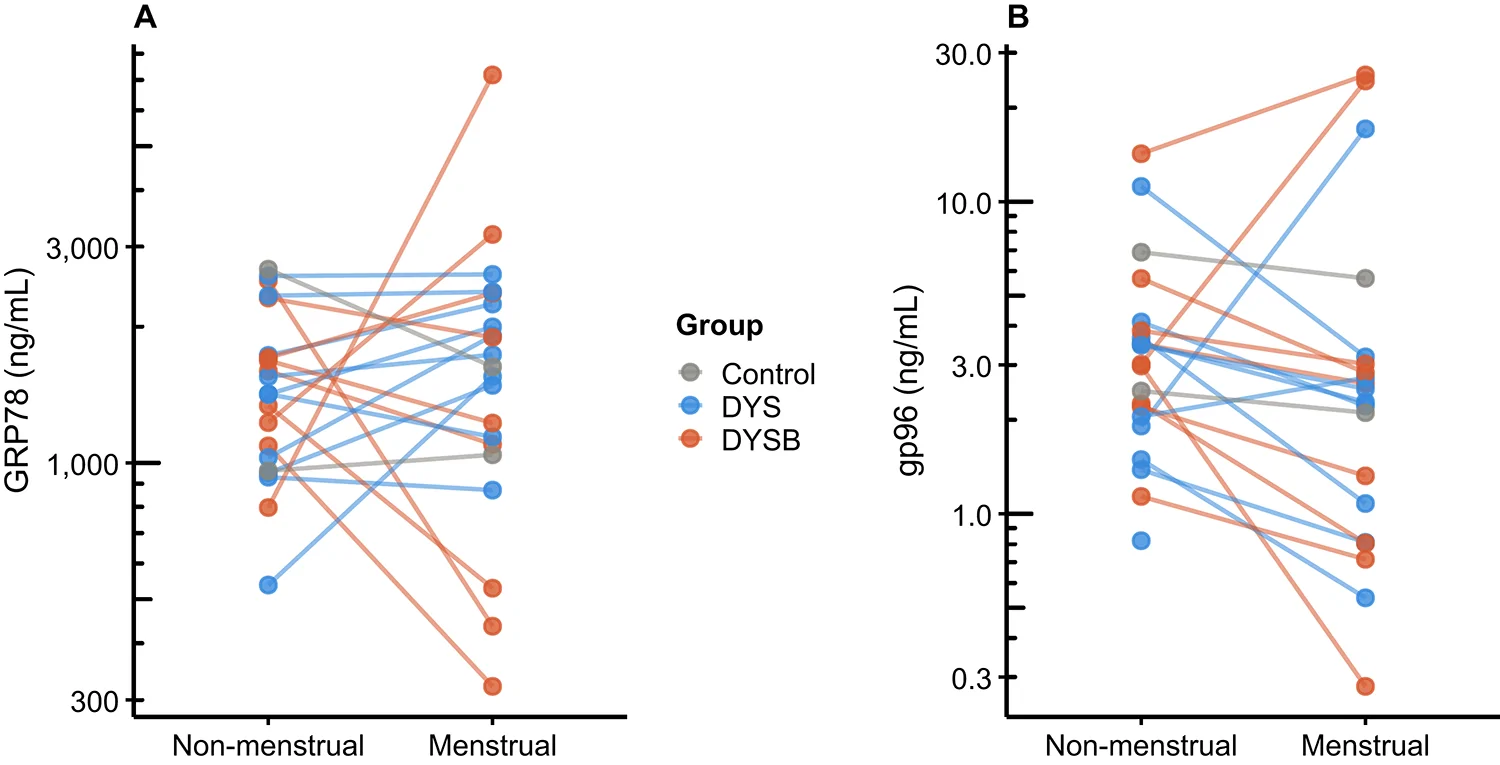
Circulating GRP78 and gp96 by menstrual phase in subjects with paired observations. Subject-level geometric means for participants with observations at both non-menstrual and menstrual time points (n = 21 paired subjects: DYS = 10, DYSB = 9, controls = 2). Lines connect within-subject observations. (**A**) GRP78 and (**B**) gp96. Neither protein showed a significant phase effect in this paired subset (mixed model, *p* > .05). In the full-sample mixed model (Table 3), gp96 was significantly lower during the menstrual phase with naproxen compared to the non-menstrual phase (*p* = .014); this effect is driven primarily by naproxen-treated visits in larger cohorts rather than by menstrual status per se. The small control paired subset (n = 2) limits visual interpretation of the control trajectory. Two GRP78 outliers (subjects 44 and 50) are excluded.

Some participants (n = 21 of 127, 17%) provided serum at both menstrual (M) and non-menstrual (NM) time points, allowing us to ask whether circulating GRP78 or gp96 vary across the menstrual cycle (Fig. 2). GRP78 was stable across phase (all phase contrasts *p* > 0.29). For gp96, levels were ~ 27% lower in menstrual samples collected under naproxen treatment than in non-menstrual samples (*p* = 0.014); menstrual samples collected without naproxen did not differ from non-menstrual samples (*p* = 0.166). A paired within-subject analysis (n = 21) showed a directionally consistent pattern (M/NM ratio 0.73, *p* = 0.106) but did not reach significance in this smaller sample. The naproxen-phase effect is addressed separately below.

### Naproxen sodium does not alter circulating GRP78 or gp96 concentrations

**Fig. 3:**
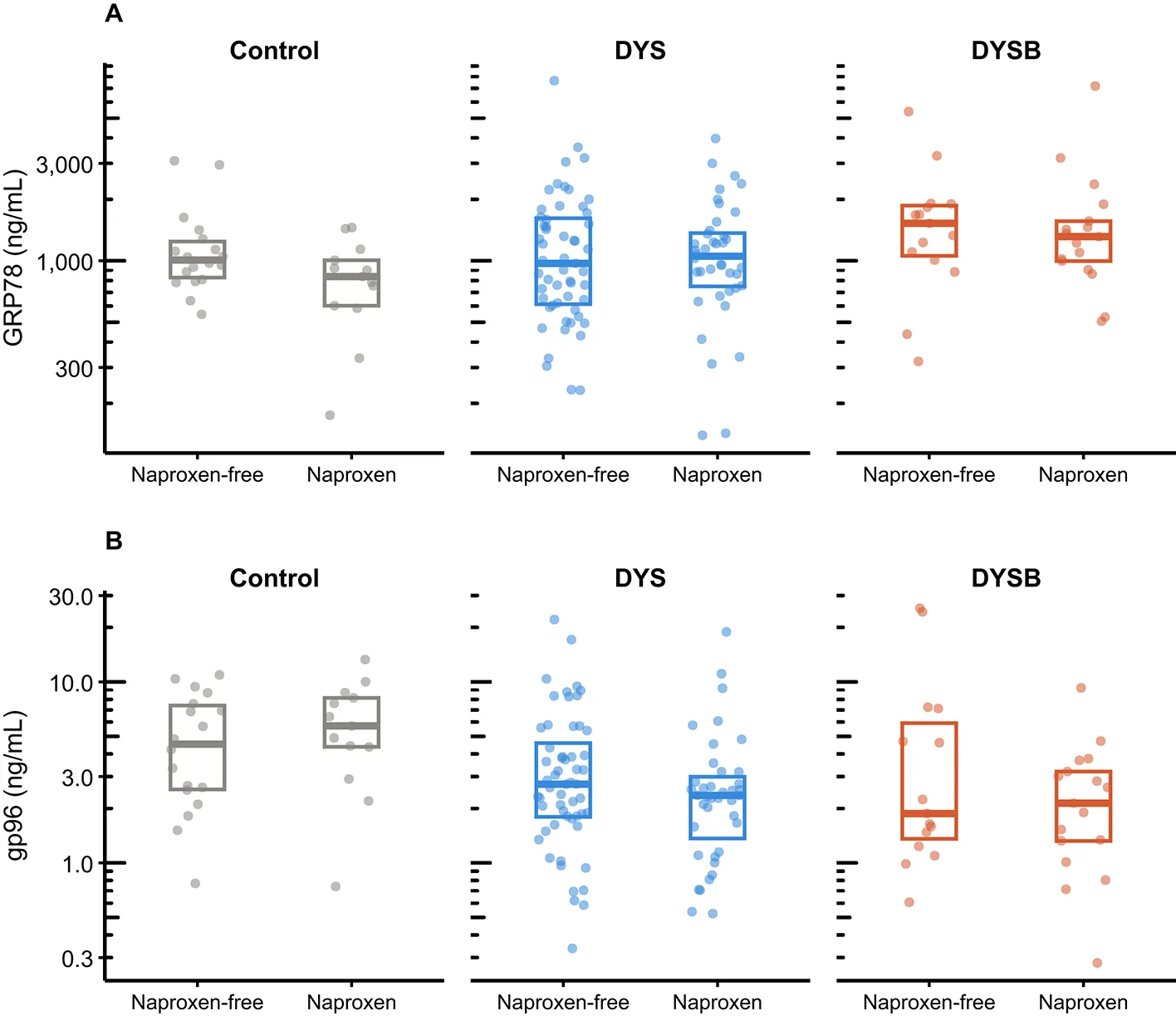
GRP78 and gp96 levels are not associated with Naproxen Sodium use during menstrual phase. Subject-level geometric means for menstrual-phase observations, stratified by naproxen administration within each group. Crossbars indicate the median with interquartile range. (**A**) GRP78 and (**B**) gp96 in controls, dysmenorrhea (DYS), and DYSB. n per group × drug cell: Control naproxen-free = 18, Control naproxen = 13; DYS naproxen-free = 56, DYS naproxen = 40; DYSB naproxen-free = 15, DYSB naproxen = 17. Within menstrual phase, direct comparisons of naproxen-free vs naproxen samples were not significant for either protein in any group. Non-menstrual observations are not included here; see Table 3 for the phase × naproxen contrasts from the full mixed model. Y-axis: log10 scale.

To see whether naproxen itself alters circulating GRP78 or gp96, we compared menstrual samples collected with vs. without naproxen exposure (Fig. 3). Direct naproxen vs naproxen-free contrasts were not significant for either protein in any group (all *p* ≥ 0.06). The apparent phase × naproxen effect on gp96 noted above (*p* = 0.014) is therefore unlikely to reflect an acute pharmacologic response.

### Associations of ER-stress proteins with symptom domains

To determine whether the group differences in GRP78 and gp96 could be explained by variation in non-menstrual pain, psychological symptoms, or demographic factors, we correlated subject-level protein levels with six clinical covariates (Table 4). After correction for multiple testing, no covariate was significantly associated with either protein. However, the negative correlation between GRP78 and PROMIS Fatigue was significant in unadjusted tests (ρ =  − 0.234, raw *p* = 0.009) but did not survive Benjamini–Hochberg correction across the 12 tests (adj. *p* = 0.107).

**Table 4:** Covariate correlations. Spearman ρ, subject-level log averages (primary dataset, subjects 44 and 50 excluded). Raw and Benjamini-Hochberg-adjusted p-values are reported for each protein. BH adjustment is applied across all 12 tests (2 proteins × 6 covariates). Significance markers reflect the value in their column. ** *p* < .01.

Covariate^a^	GRP78 ρ	GRP78 p	GRP78 p (adj.)	gp96 ρ	gp96 p	gp96 p (adj.)
PROMIS anxiety	− 0.076	0.403	0.691	0.107	0.236	0.565
PROMIS sleep disturbance	− 0.083	0.362	0.691	0.065	0.471	0.707
PROMIS depression	− 0.148	0.101	0.520	0.057	0.530	0.707
PROMIS fatigue	− 0.234	0.009**	0.107	0.123	0.173	0.520
Painful body sites (n)	− 0.133	0.143	0.520	0.014	0.881	0.934
Age	− 0.008	0.934	0.934	− 0.040	0.658	0.790

## Discussion

In this study, we investigated whether alterations in the ER stress-UPR are associated with menstrual pain by measuring circulating levels of heat shock proteins GRP78 and gp96 in individuals with dysmenorrhea, DYSB, and controls. We found that gp96 levels were reduced in both dysmenorrhea and DYSB groups, while GRP78 was selectively elevated in DYSB. Circulating GRP78 and gp96 were not significantly affected by menstrual phase, naproxen sodium use, or psychosocial factors. While primary dysmenorrhea is traditionally attributed to elevated uterine prostaglandins with localized inflammation driving painful uterine contractions, our results are consistent with the possibility that systemic ER stress-related chaperone levels are also altered in individuals with menstrual pain, though the cellular sources and upstream drivers of these circulating changes remain to be established. The opposing changes in GRP78 and gp96 are compatible with selective rather than global ER chaperone dysregulation, with the strongest signal in those with comorbid bladder sensitivity, a group at increased risk for chronic pelvic pain.

**Fig. 4:**
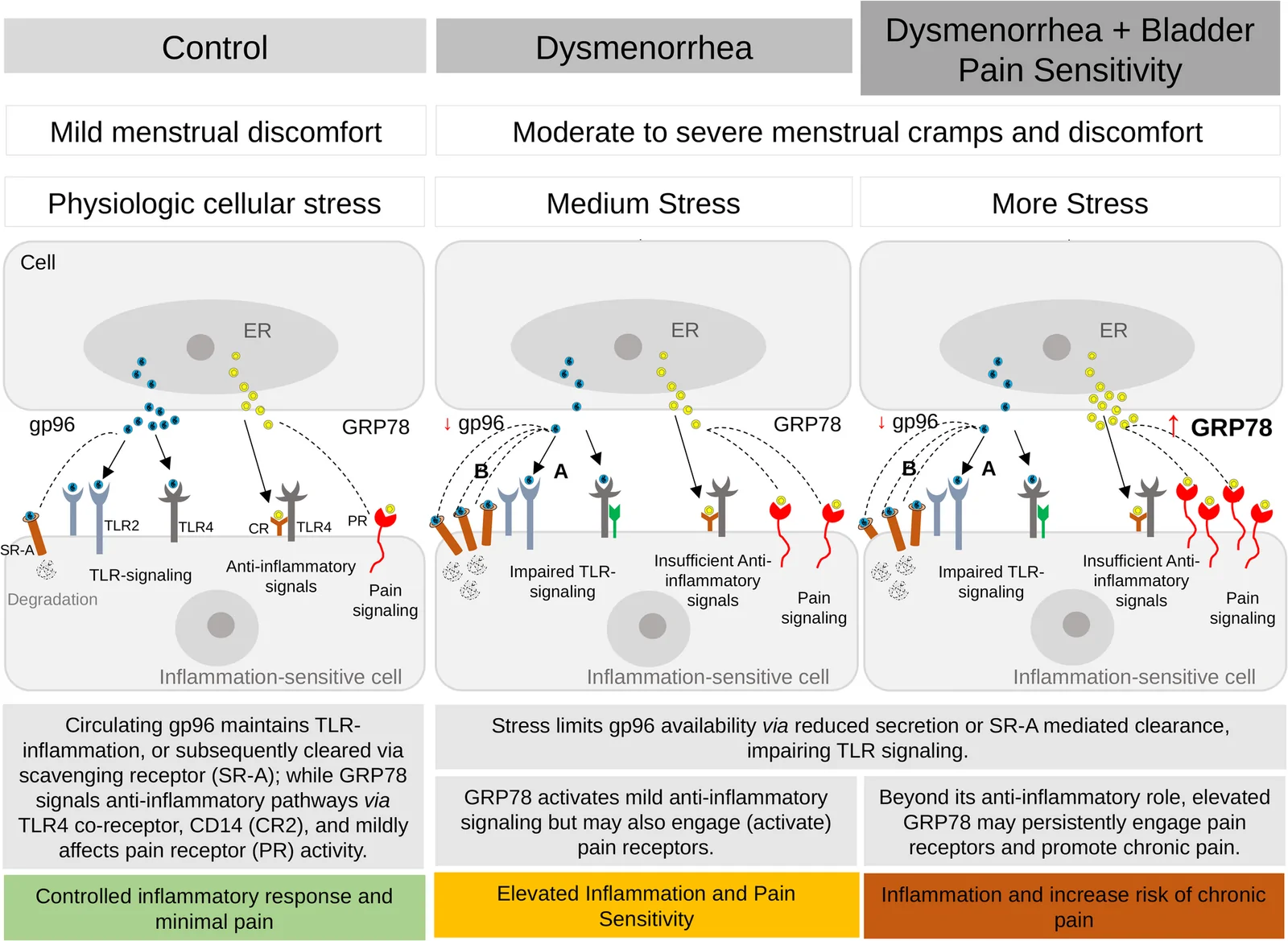
Proposed model for the role of gp96 and GRP78 in dysmenorrhea and bladder pain sensitivity. Although it is unknown whether gp96 and GRP78 play causal or downstream roles, we have hypothesized how these molecules may contribute to dysmenorrhea and bladder pain sensitivity. During a pain-free menstrual period, gp96 maintains toll-like receptor (TLR) function while GRP78 dynamically shifts between immunotolerance, immune reactivity, and pain sensitivity, resulting in a controlled inflammatory response and minimal pain. In dysmenorrhea, stress-related reduction in gp96 secretion (**A**) or its increased uptake and degradation by SR-A1 (scavenger receptors) (**B**) may alter TLR sensitivity. Concurrently, elevated GRP78 alters TLR responses to ligands that could worsen menstrual pain. In individuals with bladder pain sensitivity, GRP78 release is further elevated; whether this elevation is associated with increased chronic pain risk requires longitudinal investigation.

Variations in systemic stress parameters related to the unfolded protein response have not been a major focus in menstrual or pelvic pain to this point. While traditionally considered intracellular chaperones, growing evidence indicates that many HSPs, including GRP78 and gp96, can translocate to the cell surface or be secreted into the extracellular space and blood circulation^45,67^. These extracellular HSPs have been increasingly recognized for their broader roles in immune regulation and in the pathogenesis of inflammatory pain conditions^65,68,69^. Following tissue or nerve injury-including damage to glial cells-, HSPs are released into the extracellular space or circulation^70,71^, where these proteins bind to toll-like receptors (TLR) on immune cells, astrocytes and microglia^71,72^ and trigger the release of inflammatory mediators such as tumor necrosis factor-α, which in turn amplifies inflammatory pain and sensitization^73,74^. Although it is unknown whether circulating HSPs play a causal or downstream role, we have hypothesized how HSPs may elicit downstream effects increasing pain sensitivity in participants with dysmenorrhea (Fig. 4). Our recognition of altered HSP levels in participants with dysmenorrhea should encourage work to understand downstream impacts of such changes in pelvic organs and tissues.

### Comparison of our gp96 data to published literature

Within the HSP family, HSP90 proteins have been strongly implicated in inflammatory pain processing, pain sensitivity, and pain behavior^75,76^, In a mouse model of primary dysmenorrhea, uterine expression of cyclooxygenase-2, a key enzyme in prostaglandin synthesis, was found to be elevated alongside increased expression of HSP90ab1 (an isoform of HSP90B1/gp96) suggesting a potential mechanistic link between HSP90 and menstrual pain^42^. Circulating HSP90 has been reported to be elevated in individuals with fibromyalgia and correlated with musculoskeletal pain and somatic distress severity^57^. We note, however, that different HSP family members (HSP70, HSP90, GRP78, and gp96) have distinct intracellular chaperone functions, extracellular signaling roles, and receptor interactions. Findings for cytosolic HSP90 in fibromyalgia may therefore reflect related but not identical stress-response pathways from those involving the ER-resident paralog gp96 (HSP90B1). However, we observed the opposite result. a lower level of gp96 in both dysmenorrhea and DYSB participants. It is important to note most of the prior work on gp96 or its isoforms (HSP90, HSP90AB1), focused on intracellular or localized changes in expression^36,42,50,51,57,76–78^, but our study looked at systemic changes. One possibility for this finding may be differences in receptor-mediated scavenging in the setting of dysmenorrhea. Repeated exposure to inflammatory mediators (IL-1β, TNF-α, PGE₂) may upregulate TLR2/4 expression in the uterus via NF-κB signaling, providing more binding sites for extracellular HSPs^79^. In addition, gp96 is efficiently internalized by antigen-presenting cells through scavenger receptor-A (SR-A), CD91/low-density lipoprotein receptor-related protein 1 (LRP1), and scavenger receptor expressed by endothelial cells-I (SREC-I), limiting its persistence in circulation^80^. Therefore, we hypothesize reduced circulating gp96 in dysmenorrhea may reflect either a differential cellular stress response or increased immune receptor activity that accelerates gp96 clearance, distinguishing affected participants from controls. We emphasize that this receptor-mediated clearance mechanism is hypothesized based on evidence from other inflammatory and immune conditions and has not been directly demonstrated in dysmenorrhea; mechanistic studies will be required to establish whether SR-A, CD91/LRP1, or SREC-I pathways are differentially active in this context. Although it seems surprising gp96 is reduced in participants with dysmenorrhea, only a handful of studies have measured circulating gp96^39,57,69^.

### Comparison of our GRP78 data to published literature

GRP78 is a better-characterized ER chaperone that has received considerable attention for its role in the progression of various diseases, many of which share common features of chronic inflammation and pain^46,81,82^. Although elevated circulating GRP78 levels have been reported in several conditions, including Covid-19 infection^83^, coronary heart disease^84^, metabolic disorders^65^, and major depressive disorder^85^, reduced levels have been observed in patients with obstructive and restrictive pulmonary disorders^86^ and type 2 diabetic kidney disease^87^. These findings suggest that GRP78 release into circulation is context-dependent, varying with the type of physiological stress and underlying pathology. Consistent with prior reports linking circulating GRP78 to metabolic disease^64,65^, BMI was an independent predictor of GRP78 in our primary model (β = 0.034 per kg/m^2^, *p* = 0.009). The GRP78 elevation in DYSB persisted after adjusting for BMI, indicating that the bladder sensitivity association is not explained by adiposity. Despite its emerging clinical relevance, direct evidence linking GRP78 to the development and maintenance of pain conditions remains limited^88–92^. In the present study, we report for the first-time elevated circulating GRP78 levels in individuals with the DYSB phenotype compared to both individuals with dysmenorrhea alone and controls. Although the precise mechanisms underlying this differential elevation remain unclear, the elevated GRP78 may be influenced by circulating sex hormone levels, particularly estrogens^93,94^. This interpretation is supported by our previous findings demonstrating that individuals with DYSB exhibit significantly higher circulating estradiol levels than controls^95^. A synergistic effect of elevated estradiol and GRP78 has been shown to relate to aggressiveness of papillary thyroid cancer^78^. In light of the fact that individuals with DYSB appear at heightened risk for developing chronic pelvic pain^12^, these cross-sectional findings suggest the hypothesis that higher GRP78 could be a candidate marker associated with chronic pelvic pain risk^88–91^, particularly in concert with sex steroids. Because the present study is cross-sectional, these findings should be considered hypothesis-generating; longitudinal studies will be required to determine whether higher GRP78 carries predictive value for chronic pain emergence.

### Understanding the inverse relationship between gp96 and GRP78

We hypothesize the inverse relationship we observed, elevated GRP78 alongside reduced gp96, reflects two complementary processes (Fig. 4). First, biased activation of the unfolded protein response in dysmenorrhea could preferentially induce GRP78, which is the canonical ER stress sentinel robustly upregulated by PERK–ATF4 and IRE1–XBP1 signaling^96^. Second, extracellular gp96 may be selectively depleted through the receptor-mediated clearance pathways described above^80^. In the context of menstrual inflammation, where prostaglandins and cytokines recruit and activate innate immune cells^97^, heightened expression of these receptors could accelerate gp96 uptake and explain its reduced circulating levels. Notably, the systemic concentration gp96 is orders of magnitude lower than GRP78, making it more vulnerable to depletion. Together, this dual mechanism provides a plausible explanation for the divergent serum profile of increased GRP78 and decreased gp96 in participants with dysmenorrhea. With the exception of lower gp96 levels in menstrual samples collected under naproxen (an effect that did not survive as a direct pharmacologic contrast and should be read cautiously), neither protein showed clear sensitivity to menstrual phase, naproxen, or psychosocial factors. Given the small, heterogeneous paired subset, we interpret these largely null patterns as the absence of a detectable effect within the limits of the available sample, rather than as evidence of stability or of no effect.

### Strengths and limitations

This study provides novel observations of differential expression of circulating ER stress-related proteins GRP78 and gp96 in individuals with dysmenorrhea, particularly in a high-risk bladder-sensitive subtype. Several strengths enhance the rigor and relevance of these findings. First, the inclusion of three independent but complementary cohorts enhance generalizability across study contexts. Second, the use of validated ELISA assays with low intra- and inter-assay variability strengthens the reliability of the biomarker measurements. Finally, stratification by pain phenotype (dysmenorrhea vs. DYSB) provided greater resolution into potentially divergent pathophysiologic mechanisms, shedding some initial light on visceral pain conditions that have been historically understudied^98^. However, several limitations should be noted. The cross-sectional design precludes causal inference or assessment of biomarker trajectories over time. Circulating GRP78 and gp96 may reflect systemic processes, but their cellular sources remain unclear. Finally, although internal consistency was observed across cohorts, the absence of an external validation sample limits generalizability. Several additional limitations warrant mention. First, although participants were screened for known pelvic pathology (endometriosis, adenomyosis, and fibroids), definitive imaging or laparoscopic confirmation was not obtained in every participant; subclinical endometriosis therefore cannot be entirely excluded in the dysmenorrhea or DYSB groups. Second, GRP78 values in Study #2 (EH-19–040) were systematically lower than in the other cohorts, consistent with a pre-analytical or assay batch effect; sensitivity analyses using within-study z-scores (Supplementary Table S3) confirmed the direction of the bladder phenotype effect, but measurement heterogeneity could attenuate or bias absolute GRP78 estimates. Third, the inter-assay CV of ~ 15% for gp96, while within commercial ELISA norms for low-abundance circulating proteins, may attenuate detection of small effects. Fourth, unequal group sizes may limit precision and power for control-anchored contrasts. Despite these limitations, the higher GRP78 in DYSB and the lower gp96 in both pain groups were directionally consistent across the contributing cohorts, and within-study z-score sensitivity analyses (Supplementary Table S3) confirmed the gp96 effects (DYS vs Control *p* = 0.032, DYSB vs Control *p* = 0.045), supporting the robustness of the primary gp96 findings. The GRP78 DYSB effect was directionally consistent in the z-score analysis (*p* = 0.054) but should be interpreted as preliminary and hypothesis-generating, as it became borderline in the sensitivity analysis and the derived DYSB vs Control contrast did not reach significance. Accordingly, the gp96 findings can be described with greater confidence, whereas the GRP78 DYSB effect requires confirmation in larger independent samples.

## Conclusion

Our findings provide new insight into the role of systemic heat shock responses in dysmenorrhea and a high-risk bladder-sensitive subtype (DYSB). The opposing directions of gp96 and GRP78 in DYSB point to selective, rather than uniform, ER chaperone dysregulation in a phenotype previously linked to chronic pain risk. These cross-sectional findings identify GRP78 and gp96 as candidate circulating markers associated with dysmenorrhea-related pain phenotypes. Whether these proteins carry predictive value for chronic pelvic pain or pathophysiological relevance requires longitudinal validation and integrated mechanistic studies with tissue-specific analyses.

## Supplementary Information

Below is the link to the electronic supplementary material.

Supplementary Material 1

## Data Availability

The datasets generated during and/or analysed during the current study are available in the Open Science Framework repository, [https://doi.org/10.17605/OSF.IO/EP3Y2].
