# Supplementary material for: Expression of circulating GRP78 and gp96, endoplasmic reticulum stress-related proteins, in menstrual and bladder pain sensitivity
Source: Sci Rep. 2026 Jun 30;16:29963. doi: 10.1038/s41598-026-58824-0 (PMC13616735; doi:10.1038/s41598-026-58824-0)
Supplement: Supplementary file 1 [file 41598_2026_58824_MOESM1_ESM.docx]

**Supplementary Information**

*Expression of Circulating GRP78 and gp96 Suggests Endoplasmic Reticulum Stress in Menstrual and Bladder Pain*

**Contents**

Supplementary Figure S1. Flowchart of three independent study cohorts.

Supplementary Figure S2. Inverse correlation between circulating GRP78 and gp96.

Supplementary Table S1. Summary of visit timing, menstrual phase, and NSAID exposure across cohorts.

Supplementary Table S2. Demographic and clinical characteristics of participants across study cohorts.

Supplementary Table S3. Within-study z-score sensitivity analysis.

**Supplementary Figure S1. Flowchart of three independent study cohorts, designs and treatment protocols, and blood samples used in heat shock protein analyses.**

***Study cohort 2*:**

Mechanistic characterization of uterine pain (MCUP) to improve diagnosis and treatment for dysmenorrhea.

(n=73)

Study visits were scheduled during menses and the mid-luteal phase (16-25 days after the onset of menses). Blood samples were collected at each visit.

Study visits occurred during early menses*. NSAID regimen*: On the day of visit participants were randomized to receive either a single dose of naproxen sodium (550 mg) or a placebo. Blood samples were collected 90 minutes following administration of the assigned treatment.

Study visits occurred during non-menses phase and at menses. *NSAID regimen:* Participants were instructed to take one naproxen tablet (500 mg) every 12 hours, beginning the day before their anticipated onset of menses and continuing for the next 3 days of their period. While menstruating, participants provided blood samples at a follow-up visit scheduled between 6-8 months after study initiation. Blood was collected 90 min after ingestion of a naproxen sodium tablet.

***Study cohort 1*:**

Noninvasive imaging of uterine physiology to improve treatment for dysmenorrhea.

(n=33)

***Study cohort 3*:**

Examining the role of improved NSAID management in treating dysmenorrhea.

(n=21)

Blood samples analyzed in this study (n=127)

Schematic overview of the three contributing study cohorts. Study #1 (EH-16-263, n=33): noninvasive uterine imaging study; participants were instructed to abstain from all pain medications for at least 8 hours prior to visits. Study visits were scheduled during menses and during the non-menstrual phase (16–25 days after menses onset); blood samples were collected at each visit. Study #2 (EH-19-040, n=73): mechanistic characterization of uterine pain (MCUP) study; visits during menses with randomization to a single dose of naproxen sodium (550 mg) or placebo; blood samples were collected 90 minutes following treatment. Study #3 (EH-18-128, n=21): improved-NSAID-management dysmenorrhea study; participants took naproxen 500 mg every 12 hours starting the day before anticipated menses and continuing for 3 days; blood samples were collected during menstruation 90 minutes after a naproxen tablet, and at a non-menstrual follow-up visit 6–8 months after study initiation while on medication. Across all cohorts, 127 unique participants contributed serum samples; some samples were excluded due to hemolysis or centrifugation protocol deviations.

**Supplementary Figure S2. Inverse correlation between circulating GRP78 and gp96.**

Scatter plot of circulating GRP78 versus gp96 (subject-level log‐averaged values, both proteins on log₁₀ axes) across all participants with paired measurements (n=124). Group color indicates Control (gray), DYS (blue), and DYSB (orange). Dashed line shows the linear regression on log-transformed values; shaded ribbon is the 95% confidence interval. Spearman ρ = −0.327, p < .001. Two GRP78 outliers (subjects 44 and 50, |z|>3 in the primary GRP78 model) are excluded; gp96 includes all measurements.

**Supplementary Table S1. Summary of visit timing, menstrual phase, and NSAID exposure across the three contributing cohorts.**

| **Cohort** | **Study / Design** | **Participants (n)** | **Visits & Menstrual Phase** | **NSAID / Drug Exposure** |
| --- | --- | --- | --- | --- |
| 1 | EH-16-263 — Noninvasive imaging of uterine physiology | 33 | Two visits: one during menses and one during the non-menstrual phase (16–25 days after menses onset) | Participants instructed to abstain from all pain medications for ≥8 hours prior to each visit |
| 2 | EH-19-040 — Mechanistic Characterization of Uterine Pain (MCUP) | 73 | Single visit during early menses; randomized to drug or placebo within visit | Single oral dose of naproxen sodium 550 mg or placebo; blood drawn 90 min post-administration |
| 3 | EH-18-128 — Improved NSAID management for dysmenorrhea | 21 | Menstrual visit during anticipated menses; non-menstrual follow-up visit 6–8 months after study initiation | Naproxen 500 mg every 12 hours starting day before menses and for 3 days of period; blood drawn 90 min after a naproxen tablet during both visits |

Across all three cohorts, the same noninvasive bladder distension protocol was used to classify participants into the dysmenorrhea-with-bladder-pain-sensitivity (DYSB) phenotype based on a bladder pain rating greater than 15 on a 0–100 visual analog scale at first urge to void (or after a 1-hour maximum). All blood samples were collected via antecubital venipuncture into serum-separating tubes, centrifuged at 3,000 rpm for 10 min within 120 min of collection, and stored at −80°C until ELISA analysis.

**Supplementary Table S2. Demographic and clinical characteristics of participants across study cohorts.**

| **Study Cohort** | **1 (EH-16-263)**  **n=33** | **2 (EH-19-040)**  **n=73** | **3 (EH-18-128)**  **n=21** |
| --- | --- | --- | --- |
| Age (years) | 23 [20, 30] | 26 [21, 31] | 24 [20, 27] |
| Race  Asian  Black  Multiple Race  Other  White  Missing | 6 (18.2%)  6 (18.2%)  0 (0%)  0 (0%)  21 (63.6%)  0 (0%) | 8 (11.0%)  8 (11.0%)  6 (8.2%)  2 (2.7%)  48 (65.8%)  1 (1.4%) | 1 (4.8%)  5 (23.8%)  1 (4.8%)  0 (0%)  14 (66.7%)  0 (0%) |
| Hispanic ethnicity | 1 (3.0%) | 20 (27.4%) | 4 (19.0%) |
| Smoking history  No  Not currently  Yes | 31 (93.9%)  2 (6.1%)  0 (0%) | 69 (94.5%)  1 (1.4%)  3 (4.1%) | 18 (85.7%)  2 (9.5%)  1 (4.8%) |
| Alcohol consumption  No  Not currently  Yes | 10 (30.3%)  1 (3.0%)  22 (66.7%) | 20 (27.4%)  7 (9.6%)  46 (63.0%) | 4 (19.0%)  0 (0%)  17 (81.0%) |
| Average menstrual pain (no NSAID, 0–100 VAS) | 70 [50, 80] | 71 [60, 80] | 76 [67, 83] |
| Pain with NSAID use (0–100 VAS) | 50 [20, 60] | 39 [20, 60] | 40 [31, 43] |
| Days of missed work/school (past 90 days) | 1 [0, 3] | 2 [0, 3] | 1.5 [0, 3] |
| Menstrual cycle regularity  Always regular  Sometimes irregular  Always irregular | 26 (78.8%)  5 (15.2%)  2 (6.1%) | 62 (84.9%)  9 (12.3%)  2 (2.7%) | 20 (95.2%)  1 (4.8%)  0 (0%) |
| Oral contraceptive (current use, No) | 27 (81.8%) | 54 (74.0%) | 18 (85.7%) |
| Diagnosed medical conditions  Chronic pelvic pain  Diabetes  Inflammatory bowel disease  Migraine / headache | 1 (3.6%)  0 (0%)  0 (0%)  1 (3.6%) | 2 (2.9%)  1 (1.4%)  1 (1.4%)  6 (8.2%) | 0 (0%)  0 (0%)  0 (0%)  2 (10.5%) |
| Body Mass Index (kg/m²) | 25.4 [22.4, 28.7] | 23.4 [20.9, 26.9] | 24.2 [20.8, 27.3] |

Data presented as median [IQR] for continuous variables and n (%) for categorical variables. Cohort identifiers correspond to the IRB study protocols listed in Table S1. Abbreviations: NSAID, non-steroidal anti-inflammatory drug; VAS, Visual Analog Scale.

**Supplementary Table S3. Within-study z-score sensitivity analysis.**

***GRP78 (subjects 44 and 50 excluded before z-scoring)***

| **Parameter** | **β (SD)** | **SE** | **t** | **p** |  | **95% CI (SD)** |
| --- | --- | --- | --- | --- | --- | --- |
| DYS/B vs Control | 0.013 | 0.222 | 0.06 | 0.955 |  | [−0.422, +0.448] |
| Bladder: DYSB vs DYS | 0.375 | 0.193 | 1.94 | 0.054 |  | [−0.003, +0.753] |
| Derived: DYSB vs Control | 0.388 | 0.260 | 1.49 | 0.138 |  | [−0.122, +0.897] |
| Phase: M (naproxen-free) vs NM | 0.228 | 0.196 | 1.16 | 0.247 |  | [−0.157, +0.613] |
| Phase: M (naproxen) vs NM | 0.243 | 0.192 | 1.27 | 0.207 |  | [−0.134, +0.621] |
| OC use: Yes vs No | 0.161 | 0.192 | 0.84 | 0.404 |  | [−0.216, +0.538] |
| BMI (per kg/m²) | 0.045 | 0.019 | 2.34 | 0.020 | * | [+0.007, +0.083] |

***gp96 (full sample)***

| **Parameter** | **β (SD)** | **SE** | **t** | **p** |  | **95% CI (SD)** |
| --- | --- | --- | --- | --- | --- | --- |
| DYS/B vs Control | −0.510 | 0.236 | −2.16 | 0.032 | * | [−0.974, −0.047] |
| Bladder: DYSB vs DYS | −0.053 | 0.208 | −0.25 | 0.801 |  | [−0.460, +0.355] |
| Derived: DYSB vs Control | −0.563 | 0.279 | −2.02 | 0.045 | * | [−1.109, −0.016] |
| Phase: M (naproxen-free) vs NM | −0.436 | 0.163 | −2.67 | 0.008 | ** | [−0.756, −0.116] |
| Phase: M (naproxen) vs NM | −0.558 | 0.153 | −3.65 | <.001 | *** | [−0.858, −0.259] |
| OC use: Yes vs No | 0.295 | 0.203 | 1.46 | 0.147 |  | [−0.102, +0.693] |
| BMI (per kg/m²) | −0.023 | 0.021 | −1.11 | 0.269 |  | [−0.063, +0.017] |

Mixed-effects models on within-study z-scores of the log-transformed outcomes. Z-scores are computed within each cohort to remove between-cohort scale differences (notable for GRP78, where Study #2 / EH-19-040 shows ~2-fold lower values across all groups). Coefficients are in within-study standard-deviation units. For GRP78, z-scores are computed using means and SDs calculated AFTER excluding subjects 44 and 50 (|z|>3 in the primary GRP78 model). For gp96, z-scores are computed on the full sample (no outliers identified). DYS = dysmenorrhea without bladder pain sensitivity; DYSB = dysmenorrhea with bladder pain sensitivity; M = menses; NM = non-menses; OC = oral contraceptive. * p<.05; ** p<.01; *** p<.001.
